# Neutrophil-Lymphocyte Ratio in Predicting Infective Endocarditis: A Case-Control Retrospective Study

**DOI:** 10.1155/2020/8586418

**Published:** 2020-11-27

**Authors:** Ying Chen, Liang-jun Ye, Yue Wu, Bing-zheng Shen, Fan Zhang, Qiang Qu, Jian Qu

**Affiliations:** ^1^Department of Pharmacy, Wuhan University, Renmin Hospital, Wuhan 430060, China; ^2^Department of Pharmacy, Hunan Provincial Corps Hospital of Chinese People's Armed Police Force, Changsha 410000, China; ^3^Department of Pharmacy, Xiangya Hospital, Central South University, Changsha 410078, China; ^4^Department of Pharmacy, The Second Xiangya Hospital, Central South University; Institute of Clinical Pharmacy, Central South University, Changsha 410011, China

## Abstract

**Background:**

Infective endocarditis (IE) is a complex infectious disease with high morbidity and mortality. The inflammation mechanism of IE is a complex network including interactions of inflammatory cytokines and other components of host response. As an important inflammation marker, the prediction ability of neutrophil-to-lymphocyte ratio (NLR) in IE deserves further investigation.

**Methods:**

NLR values were measured and compared between IE patients and healthy controls, good and bad clinical outcome groups. The receiver operating characteristic curves (ROCs) of NLR and cut-off values were measured in IE patients, pathogen-subgroups, and different clinical outcome groups.

**Results:**

There were 678 IE patients and 2520 healthy controls enrolled in our study. The number of good and bad clinical outcome patients was 537 and 141, respectively. The value of NLR was significantly higher in IE patients than healthy controls (6.29 ± 9.36 vs. 1.87 ± 0.34, *p* < 0.001), and the area under the ROC (AUC) was 0.817 (95% CI (0.794, 0.839), *p* < 0.001). The critical value of NLR for diagnosis of IE was 2.68, with a sensitivity of 69%, and a specificity of 88%. The value of NLR was significantly higher in bad clinical outcome patients than in good clinical outcome patients (5.8 ± 6.02 vs. 3.62 ± 2.61, *p* < 0.001). The critical value of NLR to predict the outcome of IE was 5.557, with a sensitivity of 39.0% and a specificity of 85.3%.

**Conclusions:**

NLR is a predictive marker for IE patients, especially in Gram-negative bacteria and Gram-positive bacteria-infected IE patients. NLR also can predict the outcome of IE. Early detecting NLR upon admission may assist in early diagnosis and risk stratification of patients with IE.

## 1. Introduction

Infective endocarditis (IE) is a complex infectious disease with high morbidity and mortality [1]. The inflammation mechanism of IE is a complex network including interactions of inflammatory cytokines and other components of host response [2]. Previous studies indicated that inflammation markers such as monocyte to high-density lipoprotein cholesterol ratio (MHR) [3], Apolipoprotein A-I, HDL-C [4], and interleukin (IL)-17 [5] are favorable prognostic markers in IE. Elevated serum IL-6 and C-reactive protein (CRP) levels may suggest ongoing IE, and the former helps better and faster monitoring of treatment [6]. IL-8-containing cells in infected heart valves could be used as a marker of IE activity [7]. Rheumatoid factor (already a Duke minor criterion) is an inflammatory marker that helps in the diagnosis of patients with suspected IE [8, 9].

Leukocytes play a main role in infectious diseases [10]. As the predominant cells in white blood cells, neutrophils (NEU) and lymphocytes (LYM) carry a big weight in the inflammatory reaction of infection. Neutrophilic leukocytosis (the gradual increase in neutrophil count and the simultaneous decrease in lymphocyte count) constitute a typical leukocyte change in response to acute bacterial infections [11]. The neutrophil-to-lymphocyte ratio (NLR) can reflect, in a synergetic manner, more about disease severity than either of the former leukocyte subgroups [12]. More and more studies suggest that NLR, as an inexpensive and easily accessible inflammatory marker, is an independent predictor of unfavorable clinical outcomes in infectious [13–16] and cardiovascular diseases [17–19], subclinical diabetic cardiomyopathy, prediabetes and diabetes mellitus [20, 21], and cancers [22–24]. The relationship between admission NLR and IE was evaluated in only four studies. A retrospective study enrolled in 121 IE patients found that high NLR at admission is associated with in-hospital mortality and central neuron system events [12]. Another study confirmed it and further found that NLR showed no predictive indication of mortality with long-term follow-up [25]. While Meshaal et al. analyzed the data from 142 consecutive patients with definitive IE and found that NLR is an independent predictor of outcome in infective endocarditis; calculation of the NLR upon admission may assist in early risk stratification of patients with IE [26].

However, the relevant articles are small sample retrospective experiments, so the variety of NLR values during the entire IE process cannot be accurately predicted. Moreover, there is no comprehensive study on the IE caused by different pathogens and in different populations. Whether NLR can be used as an independent predictor of IE remains further investigation. Herein, we carried out this study to evaluate the ability of NLR to predict the occurrence and outcome of IE.

## 2. Methods

### 2.1. Study Population

The IE patients were collected from the Second Xiangya Hospital of Central South University from 2015 to 2019. Inclusion criteria follow the modified Duke criteria for the definitive diagnosis of IE [27]. The exclusion criteria were as follows: (1) patients with malignant tumor, blood system disease, receiving chemotherapy, glucocorticoid, or immunosuppressant treatment; (2) patients who were interruption of treatment or automatic discharge and no cause of death; (3) patients who had no NLR data before the treatment. Healthy individuals from the medical examination center of our hospital were enrolled in our study. In-hospital bad clinical outcome was defined and followed at least one condition: (1) hospital death; (2) any clinically overt central nervous system event, including embolic brain infarction, brain hemorrhage, transient ischemic attack, and meningitis [12]. The Ethics Committees of the Second Xiangya Hospital of Central South University approved the study protocol (yxb-lcys-201501).

### 2.2. Clinical Data Collection

The characteristic data of patients including gender, age, risk factors, blood culture results, pathogens, and body temperature were collected from the medical system. The complete blood cell counts and differential counts of leucocytes, NLR, CRP, and procalcitonin (PCT) levels were measured in the clinical laboratory department of our hospital. If the patient has multiple test results, the first one before treatment was collected in our study.

### 2.3. Statistical Analysis

Statistical analysis was performed using SPSS Statistics, version18.0 (SPSS Inc., Chicago, IL). Measured data were expressed as mean ± standard deviation (*x* ± *s*) or the median (interquartile range). The Student *t*-test was used to compare data between the groups displaying normal distribution. Nonparametric tests are used for comparison between the two groups with nonnormal distribution data. The area under the receiver operating characteristic curves (ROCs) was used to evaluate the value of NLR in predicting the prognosis of patients with IE. *p* < 0.05 was considered statistically significant.

## 3. Results

### 3.1. Characteristics

Following the inclusion criteria and exclusion criteria, we enrolled 678 patients with IE and 2520 healthy controls from the Second Xiangya Hospital during 2015 and 2019. The clinical characteristic description of the patients and healthy controls was shown in Table 1 and Table S1. The age of IE patients was 43.25 ± 17.60 years, and the age of controls was 42.28 ± 15.85 years, which had no difference (*p* = 0.86). There was also no statistical difference in sex distribution between patients and healthy controls (*p* = 0.08) (Table 1). The percentage of comorbidity and predisposing factors were 14.01% rheumatic heart disease, followed by 12.54% congenital heart disease and 5.16% sepsis. The positive rate of blood culture and cardiac valve vegetation culture was 33.92%. The pathogens of patients were 86.70% Gram-positive bacteria, 6.87% Gram-negative bacteria, and 6.43% fungus, respectively. Among the Gram-positive bacterial infections, the most common bacteria is *α-hemolytic streptococcus* (58.41%), followed by *Staphylococcus aureus* (15.35%), *Staphylococcus epidermidis* (4.46%), and *Coagulase negative staphylococci* (4.46%). Sixteen Gram-negative bacteria were isolated from culture. *Acinetobacter Baumannii*, *Escherichia coli*, and *Brucella* were the top three Gram-negative bacteria. Sixteen strains of fungi were isolated from culture, including 8 *C. parapsilosis*, 5 *Candida albicans*, and 2 *Candida glabrata*. The detailed pathogens of patients were shown in Table 1 and Table S1. The inflammatory markers of IE patients before anti-infective drug treatment including NLR, PCT, and CRP were 6.29 ± 9.36, 2.34 ± 9.53 *μ*g/L, and 53.13 ± 55.76 mg/L, respectively. NLR and NEU values of controls were significantly lower than those in IE patients (*p* < 0.001).

### 3.2. The Predictive Effect of NLR, NEU, and LYM in Patients with IE

**Figure 1:**
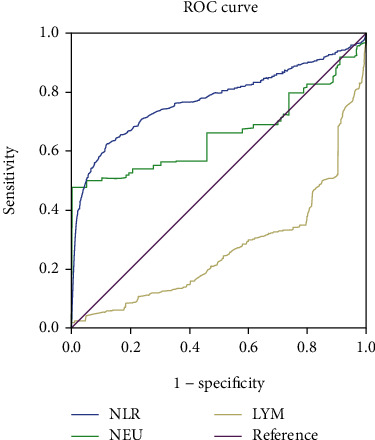
The ROC curves of NLR, NEU, and LYM for predicting all IE patients.

The ROC curves of NLR, NEU, and LYM for total IE patients were plotted (Figure 1). For all IE patients, the area under the ROC curve (AUC) of NLR was 0.817 (95% confidential interval (CI) = 0.794 − 0.839); the standard deviation was 0.012, and *p* < 0.001. The critical value of NLR for diagnosis of IE was 2.68, with a sensitivity of 69%, and a specificity of 88%. AUC of NEU and LYM was 0.66 (95% CI = 0.631 − 0.689) and 0.27 (95% CI = 0.247 − 0.293), and *p* values were both less than 0.001. The critical value of NEU for diagnosis of IE was 6.425, with a sensitivity of 47.6%, and a specificity of 97.6%. The critical value of LYM for diagnosis of IE was 5.107, with a sensitivity of 1.9%, and a specificity of 98.8% (Table 2).

### 3.3. The Predictive Effect of NLR and Other Evaluation Indexes in IE Patients with Different Culture Results

**Figure 2:**
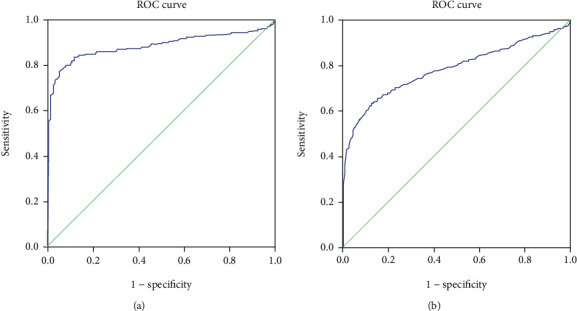
The ROC curves of NLR for predicting culture-positive IE and culture-negative IE.

In the ROC curve analysis, the critical value of NLR for diagnosis of IE with positive culture was 3.04, with a sensitivity of 79.6%, and a specificity of 92.9%; for diagnosis of IE with negative culture, the critical value was 2.675, with a sensitivity of 62.1%, and a specificity of 88.1% (Table 3, Figure 2).

**Figure 3:**
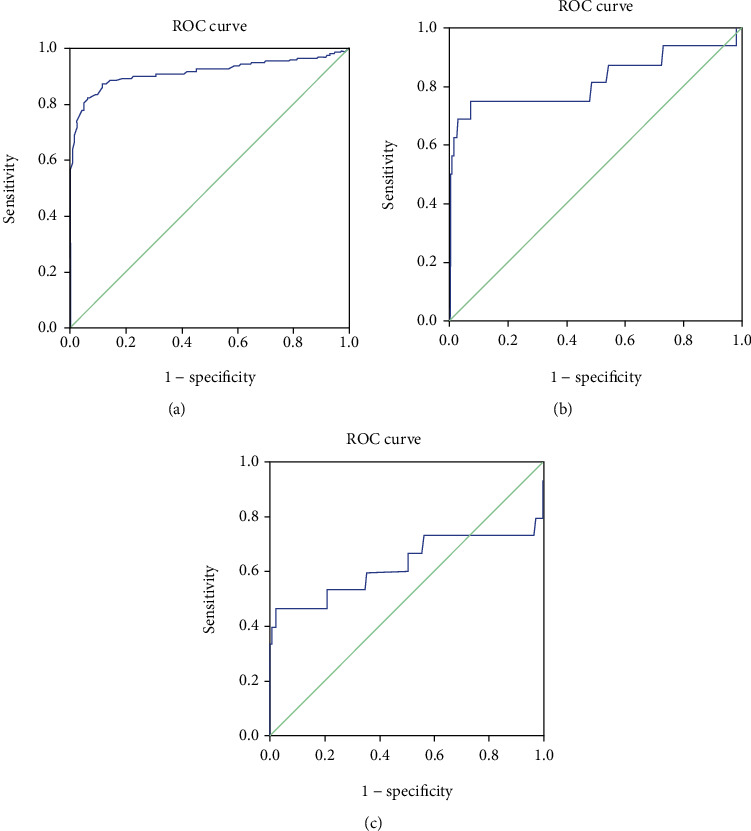
The ROC curves of NLR for predicting IE with different infectious pathogens. (a) Gram-positive bacteremia, (b) Gram-negative bacteremia, and (c) fungus.

For Gram-positive bacteremia IE patients, the ROC was 0.913 (0.882 to 0.943; *p* < 0.001), and the critical value of NLR was 3.055, with a sensitivity of 82.4%, and a specificity of 93.1%. For Gram-negative bacteremia IE, the ROC was 0.822 (0.672 to 0.973; *p* < 0.001), and the critical value of NLR was 3.035, with a sensitivity of 75%, and a specificity of 92.9%. For fungus, the ROC was 0.625 (0.416 to 0.834); the standard deviation was 0.107, and *p* = 0.096. The critical value of NLR for the diagnosis of fungi infected IE was 3.91, with a sensitivity of 46.7%, and a specificity of 97.4% (Table 3, Figure 3).

The values of CRP in IE patients with Gram-positive and Gram-negative bacteria and fungi infected were 59.28 ± 54.79, 65.98 ± 65.88, and 78.23 ± 37.41 mg/L, respectively. The value of PCT in Gram-positive bacteria-infected IE patients was 2.41 ± 8.51 *μ*g/L, which was higher than that in Gram-negative bacteria (1.42 ± 3.18 *μ*g/L) and fungi (1.86 ± 2.93 *μ*g/L), respectively (Table 4).

### 3.4. Comparison of Demographic and Laboratory Data between Different Outcome of IE Patients

**Figure 4:**
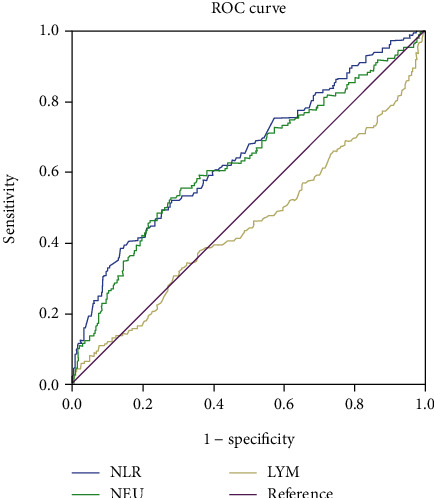
The ROC curve of NLR, NEU, and LYM for predicting good and bad outcomes of IE patients.

After evaluating the clinical in-hospital outcome of IE patients, we defined 537 good-outcome and 141 bad-outcome IE patients. There was no difference about the age, comorbid conditions, and predisposing factors between the two groups. The male distribution was higher in good-outcome patients than bad-outcome group (70.2% vs. 60.99%, *p* = 0.036). The proportion of culture-positive in good-outcome patients was less than that in bad-outcome patients (31.66% vs. 43.26%, *p* = 0.01). The proportion of fungus-infected patients in the good-outcome group was less than that in the bad-outcome group (1.12% vs. 6.38%, *p* = 0.001). And the inflammatory markers including NLR, PCT, and CRP were all different between the two groups (all *p* values were less than 0.001). NLR was higher in the bad-outcome group than that in the good-outcome group (5.8 ± 6.02 vs. 3.62 ± 2.61, *p* < 0.001) (Table 5). In the ROC curve analysis to predict the outcome of IE patients, using a cut point of 5.557, the AUC for the NLR was 0.647 (95% CI, 0.594-0.701; *p* < 0.001) with a sensitivity of 39.0% and a specificity of 85.3%. Using a cut-point of 8.095, the AUC for the NEU was 0.625 (95% CI, 0.570-0.681; *p* < 0.001) with a sensitivity of 52.5% and a specificity of 72.1%. (Figure 4, Table 2).

## 4. Discussion

Variability and atypicality in the clinical presentation of IE make the diagnosis a clinical challenge, especially for the early diagnosis [28]. Early diagnosis and effective treatment are essential to good patient outcome, which could reduce the morbidity and mortality of IE. The diagnostic strategy was currently recommended by the American Heart Association and the modified Duke criteria [27, 29]. The diagnosis of IE requires typical microorganisms grown from at least 2 separate blood cultures, which needs a relative long time [29]. Moreover, it also needs evidence of endocardial involvement, which has different symptoms due to different pathogens. Therefore, a simple blood test will help to predict IE and it is highly desirable [28].

As an inexpensive and easily accessible inflammatory marker, NLR is an independent predictor of unfavorable clinical outcomes in infectious diseases. NLR is indicative of an impaired cell-mediated immunity associated with systemic inflammation [30]. A previous study about NLR focused on its role of predicting the outcome as a simple prognostic marker [10, 12, 25]. The predictive value of the NLR is important in many tumors, infectious, and cardiovascular diseases [31–33]. In order to investigate the role of NLR in predicting the occurrence of IE, we enrolled 678 IE patients and 2520 healthy controls in our study.

To the best of our knowledge, this is the first evaluation study of NLR in predicting the early diagnosis of IE. We found that NLR values of IE patients were significantly higher than NLR values of controls. The critical value of NLR for diagnosis of IE with positive culture has a higher sensitivity and specificity than the diagnosis of IE with negative culture. The predictive effect of NLR in IE patients with Gram-positive bacteria is better than IE patients with Gram-negative bacteria. While the NLR has no predictive effect in IE patients with fungus-infected. This result implies that the predictive effect of NLR in IE depends on the culture results and different pathogens of infection. We also carried out the ROC analysis, and the cut-off values for Gram-positive, Gram-negative, and fungal pathogens are very similar, thus NLR does not seem suitable to discriminate on admission patients with IE by different pathogens. Further larger-sample clinical investigation should perform to confirm it and find other inflammatory markers to discriminate patients with IE by different pathogens.

Studies found that PCT and CRP might be valuable additional diagnostic markers in patients with suspected IE [5, 9]. One study found the area under the ROC curve that used PCT to predict IE was 0.856 (95% CI 0.750 to 0.962), compared with 0.657 (95% CI 0.511 to 0.802) for CRP [28]. Especially noteworthy is that this study compared the suspected IE patients and confirmed IE patients. Because of the lack of tests of PCT and CRP in healthy control, we did not analyze their predictive role.

Up to now, several studies have investigated the relationship between admission NLR and IE [10, 12, 25, 26]. Turak et al. showed that NLR was associated with in-hospital mortality and central nervous system events in IE patients [12]. A total of 121 IE patients were evaluated, and the study found that NLR ≥ 7.1 predicted in-hospital mortality and unfavorable outcomes [12]. Bozbay et al. also investigated 171 IE patients and found that patients in the high NLR group had a higher incidence of in-hospital mortality, while NLR cannot be a useful prognostic marker during long-term follow-up [25]. Meshaal et al. found that a higher NLR, TLC, neutrophil percentage, creatinine level, and CRP level upon admission were associated with increased in-hospital mortality and morbidity in IE patients [26]. We also evaluate the role of NLR and clinical outcome of IE patients. In our study, we defined 537 good-outcome patients and 141 bad-outcome IE patients and found NLR could predict the clinical outcome of IE to the largest extent, compared with NEU and LYM. Our relative large-sample case-control study results provide more stronger evidences that NLR is a reliable predictive biomarker of IE infection, not only in the early diagnosis but also in the outcome of IE than those already reported diseases including cerebral hemorrhage [34, 35] and ischemic stroke [36, 37].

## 5. Limitations

The limitations of our study were as follows: first, retrospective data have many uncontrollable confounding factors that limit data consistency. For instance, complicated diseases such as pneumonia and meningitis would influence the value of NLR; second, the true state of illness and treatment before their admission, such as the use of oral antibiotics before admission, also affects the accuracy of NLR value in our statistics; third, the small sample from single study center may also limit our research. Moreover, it would be more informative to evaluate mortality rather than poor clinical course, while for the limit sample-size, this study did not perform it. Further follow-up investigation is needed to conduct a mortality-related analysis.

## 6. Conclusions

In summary, our study suggests that NLR is an effective diagnostic indicator of IE. Moreover, we also found that NLR value could predict the clinical outcome of IE patients. Therefore, NLR is a useful predictive marker for IE patients. In the future, prospective studies with a larger sample-size from multicenters about the prediction ability of NLR in occurrence and clinical outcome of IE patients will be carried out to confirm our retrospective study results.

## Figures and Tables

**Table 1:** Clinical and hematologic data of study population.

Characteristics		Patients (*N* = 678)	Controls (*N* = 2520)	*p* value
Age (years)		43.25 ± 17.60	42.28 ± 15.85	0.86
Sex (man/female)		463/215	1630/890	0.08
Comorbid conditions and predisposing factors	Rheumatic heart disease	95 (14.01%)		
	Congenital heart disease	85 (12.54%)		
	Sepsis	35 (5.16%)		
	Hypertension	39 (5.75%)		
	Brain disease	9 (1.33%)		
	Coronary artery disease	74 (10.91%)		
	Nephropathy	84 (12.39%)		
	Diabetes	95 (14.01%)		
	Pulmonary infection	12 (1.77%)		
	Cardiac insufficiency	124 (18.29%)		
Pathogens (no. (%))	Culture positive	230 (33.92%)		
	Culture negative	448 (66.08%)		
	Gram-positive bacteria	202 (86.70%)		
	Gram-negative bacteria	16 (6.87%)		
	Fungus	15 (6.43%)		
Inflammatory markers	NEU	7.17 ± 4.13	5.43 ± 3.21	<0.001
	LYM	2.23 ± 1.71	1.85 ± 1.48	0.67
	NLR	6.29 ± 9.36	1.87 ± 0.34	<0.001
	PCT (*μ*g/L)	2.34 ± 9.53		
	CRP (mg/L)	53.13 ± 55.76		

NLR: neutrophil-lymphocyte ratio; PCT: procalcitonin; CRP: C-reactive protein; NEU: neutrophils; LYM: lymphocytes.

**Table 2:** The ROC and AUC data of NLR, NEU, and LYM in predicting the occurrence and outcome of IE patients.

Groups	Markers	AUC	SD	*p* value	95% CI		Cut-off	Sensitivity	Specificity	1-specificity
					Lower	Upper				
Total IE vs. normal	NLR	0.817	0.012	<0.001	0.794	0.839	2.681	0.690	0.880	0.120
	NEU	0.66	0.015	<0.001	0.631	0.689	6.425	0.476	0.976	0.024
	LYM	0.27	0.012	<0.001	0.247	0.293	5.107	0.019	0.988	0.012
Outcome good vs. bad	NLR	0.647	0.027	<0.001	0.594	0.701	5.557	0.390	0.853	0.147
	NEU	0.625	0.028	<0.001	0.570	0.681	8.095	0.525	0.721	0.279
	LYM	0.449	0.029	0.06	0.391	0.506	4.315	0.057	0.974	0.026

NLR: neutrophil-lymphocyte ratio; NEU: neutrophils; LYM: lymphocytes; AUC: the area under the ROC curve; SD: standard deviation; IE: infective endocarditis.

**Table 3:** The ROC and AUC data of NLR in predicting IE with different culture results.

NLR	AUC	SD	*p* value	95% CI		Cut-off	Sensitivity	Specificity	1-specificity
				Lower	Upper				
Culture positive vs. normal	0.888	0.017	<0.001	0.855	0.92	3.040	0.796	0.929	0.071
Culture negative vs. normal	0.782	0.105	<0.001	0.753	0.811	2.675	0.621	0.881	0.119
Gram-negative bacteria	0.822	0.077	<0.001	0.672	0.973	3.035	0.750	0.929	0.071
Gram-positive bacteria	0.913	0.015	<0.001	0.882	0.943	3.055	0.824	0.931	0.069
Fungus	0.625	0.107	0.096	0.416	0.834	3.910	0.467	0.974	0.026

ROC: receiver operating characteristic; CI: confidence intervals; SD: standard deviation; AUC: the area under the ROC curve.

**Table 4:** Infection markers in IE patients with different pathogen culture results.

Infection markers	Negative culture (*N* = 448)	Positive culture (*N* = 230)	Gram-positive bacteria (*N* = 202)	Gram-negative bacteria (*N* = 16)	Fungus (*N* = 15)	*p* value
PCT (*μ*g/L)	2.37 ± 10.40	2.30 ± 7.96	2.41 ± 8.51	1.42 ± 3.18	1.86 ± 2.93	0.766
CRP (mg/L)	49.00 ± 55.77	60.85 ± 54.91	59.28 ± 54.79	65.98 ± 65.88	78.23 ± 37.41	0.288
NLR	5.76 ± 8.61	7.93 ± 10.14	7.27 ± 6.78	13.44 ± 16.95	11.59 ± 25.39	0.021

**Table 5:** Clinical and hematologic data compared between different clinical outcomes of IE patients.

Parameters	Good outcome (*N* = 537)	Bad outcome (*N* = 141)	*p* value
Age	42.56 ± 16.81	45.89 ± 20.26	0.074
Sex (male)	377 (70.2%)	86 (60.99%)	0.036
Comorbid conditions			
Coronary artery disease	58 (10.81%)	16 (11.34%)	0.853
Nephropathy	62 (11.55%)	22 (15.6%)	0.193
Diabetes	77 (14.34%)	18 (12.76%)	0.632
Pulmonary infection	9 (1.68%)	3 (2.13%)	0.721
Cardiac insufficiency	103 (19.18%)	21 (14.89)	0.241
Hypertension	29 (5.4%)	10 (7.09%)	0.443
Brain disease	7 (1.3%)	2 (1.42%)	1
Predisposing factors			
Rheumatic heart disease	78 (14.53%)	17 (12.06%)	0.452
Congenital heart disease	68 (12.66%)	17 (12.06%)	0.847
Prosthetic valve	105 (19.55%)	28 (19.86%)	0.935
Degenerative valve disease	75 (13.97%)	14 (9.93%)	0.206
Implantable cardiac devices	32 (5.96%)	10 (7.09%)	0.619
Pathogens			
Culture positive	170 (31.66%)	61 (43.26%)	0.010
Culture negative	367 (68.24%)	80 (56.74%)	—
Gram-positive bacteria	152 (28.31%)	47 (33.33%)	0.243
Gram-negative bacteria	12 (2.23%)	5 (3.55%)	0.375
Fungus	6 (1.12%)	9 (6.38%)	0.001
Inflammatory markers			
NEU	6.84 ± 4.60	9.07 ± 5.60	<0.001
LYM	2.23 ± 1.68	2.22 ± 1.90	0.961
NLR	3.62 ± 2.61	5.80 ± 6.02	<0.001
PCT (*μ*g/L)	1.94 ± 9.67	3.15 ± 7.18	<0.001
CRP (mg/L)	50.14 ± 50.23	64.24 ± 63.02	<0.001

## Data Availability

The data used to support the findings of this study are included within the article and within the supplementary information file.

## Supplementary Materials

Supplementary Materials Table S1: the details of pathogenic microorganisms of infective endocarditis.
