# Supplementary material for: Neutrophil-Lymphocyte Ratio in Predicting Infective Endocarditis: A Case-Control Retrospective Study
Source: Mediators Inflamm. 2020 Nov 27;2020:8586418. doi: 10.1155/2020/8586418 (PMC7735837; doi:10.1155/2020/8586418)
Supplement: Supplementary Materials — Table S1: the details of pathogenic microorganisms of infective endocarditis. [file 8586418.f1.pdf]

Neutrophil-lymphocyte ratio in predicting infective endocarditis: a case-control retrospective study.

Table S1 The details of pathogenic microorganisms of infective endocarditis.

Gram-positive bacteria	N(%)	Gram-negative bacteria	N(%)	Fungus	N(%)
α-hemolytic streptococcus	118 (58.41%)	Acinetobacter Baumannii	3 (18.75%)	C.parapsilosis	8 (53.33%)
Staphylococcus aureus	31 (15.35%)	escherichia coli	3 (18.75%)	Candida albicans	5 (33.33%)
Staphylococcus epidermidis	9 (4.46%)	Brucella	2 (12.5%)	Candida glabrata	2 (13.33%)
Coagulase negative staphylococci	9 (4.46%)	Stenotrophomonas maltophilia	1 (6.25%)		
Enterococcus faecalis	5 (2.48%)	Pseudomonas aeruginosa	1 (6.25%)		

Streptococcus mitis	4 (1.98%)	Chryseobacterium indologenes	1 (6.25%)
Staphylococcus haemolyticus	4 (1.98%)	Klebsiella pneumoniae	1 (6.25%)
Streptococcus mutans	3 (1.49%)	Chryseobacterium	1 (6.25%)
Enterococcus faecium	3 (1.49%)	Enterobacter cloacae	1 (6.25%)
viridans streptococcus	2 (0.99%)	Haemophilus parainfluenzae	1 (6.25%)
streptococcus bovis	2 (0.99%)	Haemophilus influenza	1 (6.25%)
Staphylococcus hominis	2 (0.99%)		
Streptococcus milleri	1 (0.5%)		
Taphylococcus capitis	1 (0.5%)		
Streptococcus salivarius	1 (0.5%)		

Micrococcus	1 (0.5%)		
Streptococcus constellatus	1 (0.5%)		
Streptococcus sanguis	1 (0.5%)		
Group G Streptococcus	1 (0.5%)		
Group A β-haemolytic streptococcus	1 (0.5%)		
Rothia	1 (0.5%)		
Streptococcus acidominimus	1 (0.5%)		
Total	202	16	15
